# Equity in Digital Mental Health Interventions in the United States: Where to Next?

**DOI:** 10.2196/59939

**Published:** 2024-09-24

**Authors:** Athena Robinson, Megan Flom, Valerie L Forman-Hoffman, Trina Histon, Monique Levy, Alison Darcy, Toluwalase Ajayi, David C Mohr, Paul Wicks, Carolyn Greene, Robert M Montgomery

**Affiliations:** 1 Woebot Health San Francisco, CA United States; 2 Joan & Irwin Jacobs Center for Health Innovation University of California, San Diego San Diego, CA United States; 3 Center for Behavioral Intervention Technologies Northwestern University Feinberg School of Medicine Chicago, IL United States; 4 Sano Genetics London United Kingdom; 5 United States Department of Veterans Affairs Mann-Grandstaff Veterans Affairs Medical Center Spokane, WA United States

**Keywords:** Digital Mental Health Interventions, mental health, health equity, access to health care, health plan implementations

## Abstract

Health care technologies have the ability to bridge or hinder equitable care. Advocates of digital mental health interventions (DMHIs) report that such technologies are poised to reduce the documented gross health care inequities that have plagued generations of people seeking care in the United States. This is due to a multitude of factors such as their potential to revolutionize access; mitigate logistical barriers to in-person mental health care; and leverage patient inputs to formulate tailored, responsive, and personalized experiences. Although we agree with the potential of DMHIs to advance health equity, we articulate several steps essential to mobilize and sustain meaningful forward progression in this endeavor, reflecting on decades of research and learnings drawn from multiple fields of expertise and real-world experience. First, DMHI manufacturers must build diversity, equity, inclusion, and belonging (DEIB) processes into the full spectrum of product evolution itself (eg, product design, evidence generation) as well as into the fabric of internal company practices (eg, talent recruitment, communication principles, and advisory boards). Second, awareness of the DEIB efforts—or lack thereof—in DMHI research trials is needed to refine and optimize future study design for inclusivity as well as proactively address potential barriers to doing so. Trials should incorporate thoughtful, inclusive, and creative approaches to recruitment, enrollment, and measurement of social determinants of health and self-identity, as well as a prioritization of planned and exploratory analyses examining outcomes across various groups of people. Third, mental health care advocacy, research funding policies, and local and federal legislation can advance these pursuits, with directives from the US Preventive Services Taskforce, National Institutes of Health, and Food and Drug Administration applied as poignant examples. For products with artificial intelligence/machine learning, maintaining a “human in the loop” as well as prespecified and adaptive analytic frameworks to monitor and remediate potential algorithmic bias can reduce the risk of increasing inequity. Last, but certainly not least, is a call for partnership and transparency within and across ecosystems (academic, industry, payer, provider, regulatory agencies, and value-based care organizations) to reliably build health equity into real-world DMHI product deployments and evidence-generation strategies. All these considerations should also extend into the context of an equity-informed commercial strategy for DMHI manufacturers and health care organizations alike. The potential to advance health equity in innovation with DMHI is apparent. We advocate the field’s thoughtful and evergreen advancement in inclusivity, thereby redefining the mental health care experience for this generation and those to come.

## Introduction

The past few decades have marked significant momentum in the digital mental health field. More than 350,000 health-related mobile apps are available and a significant portion of these are specifically related to mental health support [1]. Enthusiasts postulate that digital mental health interventions (DMHIs) may eliminate commonplace access barriers [2,3] while also providing evidence-based mental health care that yields meaningful outcomes [4,5]. Moreover, there is a tenable undercurrent of hope, combined with appropriate and poignant questioning, on whether or not DMHI can genuinely and reliably reduce health care disparities. “Techquity” is a recently coined colloquial term that refers to this potential to either bridge or hinder equitable health care [6,7].

Problems with access to mental health care, both historical and present, are well documented across the United States and are especially pronounced within minoritized and underserved populations [8-13]. A key and increasingly common approach to understanding these inequities is through the study of social determinants of health (SDOH), which focuses on how the various circumstances in which people live affect their health and well-being [14,15]. Groups of individuals affected by systemic inequities in mental health care, in terms of both prevalence and care access, have been identified via various SDOH factors including, but not limited to, people who experience stigma or discrimination because of race, ethnicity, class, gender, sexual orientation (eg, LGBTQIA+ [Lesbian, Gay, Bisexual, Transgender, Queer, Intersex, Asexual+]); language or another aspect of identity; those with low household income or educational attainment; those who are underemployed or unemployed; older adults; those living in rural communities; members of Medicare or Medicaid; uninsured or underinsured people; veterans; and people living with disabilities [16-20]. When considering DMHIs, we must also acknowledge the “digital divide,” as many individuals may lack access, consistent connectivity, skills, or trust to engage with digital mental health technologies, further contributing to and even widening existing inequities, as was the case during the COVID-19 pandemic [21-23].

**Figure 1:**
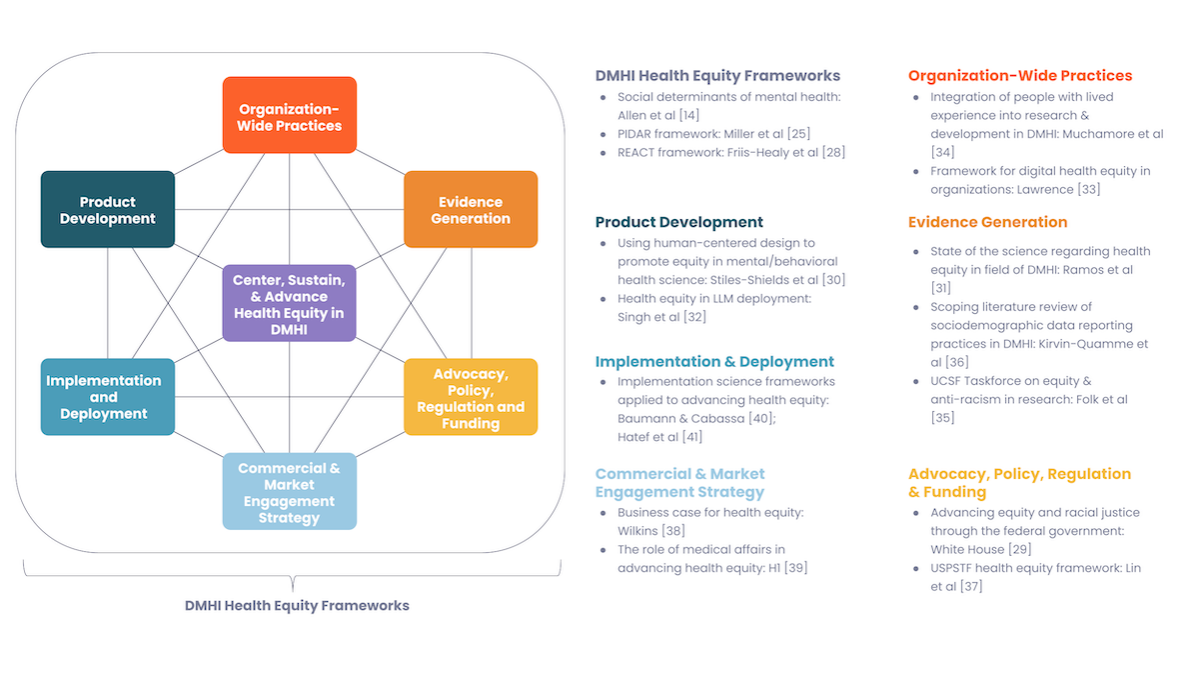
Integral elements toward building, sustaining, and advancing equitable mental health care and corresponding recommended reading. DMHI: digital mental health intervention; LLM: large language model; PIDAR: Partner, Identify, Demonstrate, Access, Report; USPSTF: US Preventive Services Taskforce.

The arc of *how* to progress toward bridging equitable health care is multifaceted, complex, dynamic, and of course, evergreen. Scholars have contributed important theoretical and applied work toward addressing inequities in digital health and DMHI, focusing on a range of topics from development to dissemination [24-28]. This viewpoint, in the spirit of the overarching special issue theme “Reflecting on Transformative Technologies, Interventions, Methods, and Policy Issues,” aims to contribute to the conversation on health equity in US-based DMHIs in 2 ways. First, by referencing extant frameworks and best practices, and second, by articulating perspectives across key stages of product and organizational maturation, from initial product development to commercialization and health care ecosystem adoption (Figure 1; see also [14,25,28-41]). The present authorship collaboration will do this by drawing upon its collective expertise in several relevant domains, including scientific research in academia and industry, DMHI commercialization and deployment strategies from both industry and health care system perspectives, DMHI product development, and organizational leadership. We also include salient illustrative examples from real-world development at Woebot Health, a US-based company that manufactures DMHIs, with which a number of the authors are affiliated.

To begin to articulate the arc, we first focus on key elements in product and intervention development. Available development frameworks call for requisite community involvement, partnership, and trust throughout as well as embedded technological support for varied digital literacy levels [25,28]. Beyond development, multiple DMHI app evaluation frameworks have been published [42-44], some with specific callouts for the import of diversity equity and inclusion therein [45]. Moreover, industry manufacturers and academicians alike are encouraged to consider their company’s or lab’s integration of diversity, equity, inclusion, and belonging (DEIB) principles into talent recruitment, workstreams, and advisory boards. Next, we discuss considerations in evidence generation. Several recent systematic reviews and meta-analyses suggest that DMHI is effective for addressing symptoms of depression and anxiety [46-48], including across the age range from adolescents [49] to older adults [50]. However, simultaneous calls to action for enhanced diversity in clinical trials underscored the ongoing need for inclusive research [29,51], calling into question whether these conclusions may be biased due to unrepresentative samples [52]. Methodological strategies to facilitate inclusive clinical trial designs will be considered. The adoption of evidence-based DMHIs has been encouraged by the American Medical Association [53] and American Psychiatric Association [54], perhaps augmenting existing options for mental health care [55]. We also highlight the need for various ecosystems (academic, industry, payer, provider, and value-based care organizations) to articulate their requirements for health equity to be a part of a manufacturer’s DMHI product and evidence-generation portfolio before adoption or deployment within a system. Specification of considerations of an equity-informed commercialization strategy for evidence-based DHMI will be discussed.

Given the promising yet relatively nascent status of the DMHI field, we encourage transparent and open conversation and a collective articulation to help specify and manifest an evergreen process of monitoring for and reducing mental health care inequities.

## Product Development

As noted in the World Health Organization’s Global Strategy on Digital Health 2020-2025, “When planning and prioritizing digital health interventions...the specific potential of digital technologies to promote health equity should be leveraged. Designed properly, digital solutions can propel inclusiveness as digital connectivity can transcend physical barriers” [56]. As any creation usually bears some imprint of the creator, a key starting principle for building an inclusive service is ensuring that the team that builds it represents a diverse set of perspectives and backgrounds, which include those of the intended users. Equity must also be baked into the product development life cycle. Similar to the European Union’s General Data Protection Regulation (GDPR) principle of “Privacy by Design,” we may think about this as “Equity by Design.” This entails explicit exploration of whether equity is likely to be achieved early on in design sessions, identifying any potentially problematic pitfalls and addressing them systematically. Human-centered design roots itself in the philosophy that thorough discovery and understanding of human needs optimize product design to meet those needs, as well as making the experience meaningful and satisfying [57]. Some human-centered design frameworks have been tailored to address DMHIs broadly [58], while others specifically target improving the health equity of DMHI [30,59]. For example, human-centered design approaches, which organically focus on inviting and hearing needs at hand, should be purposefully extended to include voices from marginalized communities [30,60], including communities with diverse digital literacy levels [61]. The PIDAR (Partner, Identify, Demonstrate, Access, Report) framework, developed by the Society of Behavioral Medicine’s Health Equity Special Interest Group, highlights partnerships as both integral to avoiding increased health inequities in DMHI and a fundamental first step in an equity-centric intervention development approach [25]. Methodologies for community-based participatory research have been articulated and incorporate strategies such as rapid prototyping and iteration for efficient colearning, as well as key human-centric and empathy-first principles of commitment, collaboration, and listening [62-64].

As more DMHIs begin to integrate aspects of artificial intelligence (AI), machine learning (ML), and large language models (LLMs), issues surrounding health equity in the rapidly evolving world of AI/ML are particularly salient to this conversation. These technologies hold great potential to, for example, enhance a conversational agent’s ability to understand natural language inputs as well as generate nuanced and tailored responses. However, there are a number of risks associated with the use of LLMs in mental health, both general and specific to health equity concerns. General concerns are LLM’s potential to “hallucinate” or generate false or misleading information as well as the limitations imposed by training data, which can be biased, inaccurate, or even actively harmful [65]. Equity-related LLM risks include their potential to exacerbate or amplify disparities if algorithms are constructed using data that reflect historical or societal biases and inequities; additionally, they increase skepticism and alienation of minoritized individuals due to lack of transparency and perception of bias [66,67]. Furthermore, many LLMs are trained primarily on a corpus of English sources, and may only be available in certain languages (eg, English), creating potential biases and risks for nonprimary language learners in understanding and interpreting these interventions, or directly excluding large swaths of people who do not speak certain languages [31,68,69]. Health equity–centered AI development frameworks are emerging to proactively address such risks [70-72]. For example, frameworks focused on the integration of LLMs into health care products recommend that LLMs are (1) thoroughly evaluated for equity-related risks, (2) rigorously monitored during deployment, (3) integrated into systems with humans-in-the-loop (eg, providers or users) to guard against biased algorithmic drift, and (4) trained in collaboration with impacted communities or populations they are intended for, all of which can help mitigate the exacerbation of potential biases [32,61,72]. Certainly, more research as well as the development and refinement of regulatory frameworks for responsible AI utilization in DMHI is called for overall [73]. President Biden’s Executive Order highlights the need to protect the “rights and safety of the public” through (1) strengthening AI governance, (2) advancing responsible AI innovation, and (3) managing risk from the use of AI [74].

## Organizationwide Practices

Embedding DEIB practices into the fabric of organizations, from corporate objectives to hiring practices and more, has been advocated for openly, and is another key element of the larger techquity conversation with implications for DMHIs [33,75]. There is strong support for the benefits that a diverse workforce can bring to an organization in terms of key business outcomes (eg, innovation and revenue generation), as well as aligning with what talent may seek within a company’s culture [76,77]. For mental health–focused organizations, the inclusion of people with lived experience of mental health challenges is especially critical, as their involvement in all aspects of DMHI research, development, policy, and practice is fundamental to promoting equity [34,78]. Diversity at both the inception of digital health innovation (eg, within design teams at DMHI manufacturing organizations) and in health care systems deploying such innovations (eg, accountable care organizations or integrated delivery networks) is part of the holistic picture [33]. Organizational commitment to various endeavors and working groups (eg, podcasts, blogs, hiring practices, educational seminars, advisory boards, health benefits, and employee well-being programs, task forces) that host and elevate conversations on equity promote recognition of its fundamental importance as well as set a regular cadence of expected updates or outputs [79-82]. Examples are the DMHI industry commitment to supporting their own employees’ mental health via a variety of DEIB efforts (ie, Headspace [83]), the establishment of a clinical diversity advisory board (ie, Woebot Health [80]), and podcast interviews with industry leaders to discuss key DEIB topics (ie, Meeting of the Minds Podcast; Health Nonprofit Digital Marketing [82,84]). Powerful work led by the University of California San Francisco’s Taskforce on Equity and Anti-Racism in Research underscored several core learnings from their endeavors, among them a clear call for a diverse workforce across the full membership of the research team, as well as ongoing education and support for DEIB in research [35]. The National Institutes of Health (NIH) offers awards dedicated to engaging undergraduate students from diverse backgrounds in biomedical research [85,86] and Rush Education and Career Center Hub (REACH) offers support in STEM (Science, Technology, Engineering, Mathematics) research to students from preschool age onward [87]. Both of these highlight direct efforts to diversify researchers of the future.

## Evidence Generation

### Enhancing Health Equity in Evidence Generation for DMHIs: Frameworks and Methodological Considerations

The generation of evidence of the feasibility, acceptability, efficacy, effectiveness, and safety of DMHIs provides another critical opportunity for the consideration of health equity. Existing frameworks that have been used to explore the centering of health equity in research are PIDAR, RE-AIM (Reach, Effectiveness, Adoption, Implementation, Maintenance), and others, many of which highlight partnerships with diverse stakeholders and potential users, consideration of how access to technology and digital literacy affect eligibility, and how data are collected and disseminated [25,26,31]. In addition to these frameworks, we offer further methodological considerations to center evidence generation in health equity, drawing on our experience in implementing these practices.

Meta-analytic research has generally supported DMHI’s efficacy, feasibility, and acceptability [46-48]. However, despite the anticipated promise of DMHIs in addressing the health equity gap, little is known about outcomes across various sociodemographic subgroups and whether the trials recruited diverse samples; additionally, only a few studies have specifically focused on interventions intended for minoritized populations [31,88]. For example, a recent systematic literature review spearheaded by Woebot Health highlighted the underreporting of sociodemographic characteristics in clinical trials evaluating DMHIs and pointed to even fewer reporting any results by sociodemographic characteristics [36]. To examine the real impact of DMHIs on health equity, evidence-generation methodology needs to be thoughtfully designed and implemented.

### Recruitment and Trial Design

First, the recruitment of diverse samples is the foundation upon which evidence generation rests. A variety of tactics can be used to recruit diverse samples, including the use of culturally sensitive recruitment materials; community outreach; thoughtful partnerships with socially, economically, geographically, or racially diverse health care systems; resources to support those with limited access to the internet; and diverse research and recruitment teams [25,28]. For example, in a recent research collaboration between Scripps Translational Science Institute and Woebot Health, utilization of such methods facilitated successful recruitment and enrollment of the a priori target of approximately 50% of a sample self-identifying as individuals from categories noted as historically underrepresented in biomedical research, including racial/ethnic minorities and rural/nonmetropolitan area residents, among others [89]. Real-world partnerships are particularly important for inviting collaboration with marginalized groups and exploring the potential of the DMHI to address health equity, with the necessity of real-world evidence outlined as an important component in several frameworks on health equity within DMHIs [26,28,31]. In these cases, clinician-referred recruitment should be managed with care and not utilized as a stand-alone recruitment strategy. Medical research has shown that clinicians and researchers may selectively exclude certain minority participants due to biases or assumptions about their interest in research, ability to follow study protocols, and likelihood of dropping out, among others [90]. Indeed, a large review [91] of minority participation in health research demonstrated that while minorities are willing to participate, they are less likely to be recruited. Where possible, health systems, patient registries, or other common recruitment sources should consider an “opt-out” method for study recruitment invitations. Specifically, upon entry to the system/registry, patients consent by default to be contacted for research opportunities unless they choose to “opt out” of such communications. This might allow for a greater diversity of individuals to be invited to participate in studies in the first place, without compromising their agency to decide which particular studies to apply for. To guide recruitment, specific targets for race, ethnicity, sexual orientation, and gender, among others, should be determined a priori based on the desired end users and relevant prevalence rates. While attempting to create targets based on census data or population prevalence is one viable option, we suggest aligning targets more closely with the prevalence rates of mental health problems in specific sociodemographic groups to more accurately capture real-world needs [92]. Additionally, to have a sufficient sample size to examine group differences, recruiting large enough samples or overrecruiting for a particular group(s) of interest may be necessary.

### Sociodemographic Surveys and Data Collection

Second, sociodemographic surveys need to be thoughtfully designed and consistently implemented. Sociodemographic reporting should include culturally sensitive and inclusive response options to questions on race, ethnicity, sexual orientation, and gender identity, as well as questions related to SDOH such as food and housing insecurity [93]. To promote honest disclosure, participants should be given the opportunity to respond electronically and be assured of the privacy and protection of their sensitive data. Of note, it is often more challenging to collect comprehensive sociodemographic and SDOH data in the context of deployment outside of research settings. Thoughtful approaches to the collection and use of these data should be agreed upon by stakeholders and tested with end users to ensure that factors such as assessment burden, privacy concerns, and potential cultural differences in the appropriateness of questionnaire content are appropriately considered.

### Analysis and Outcomes

Third, analyzing and reporting on sociodemographic characteristics and outcomes across subgroups are integral to examining the impact of DMHIs on health equity. At a minimum, DMHI research should provide the sociodemographic characteristics of their sample. Additionally, consumer-based DMHI data (outside the research context) should be presented in dashboards and reports that include a breakdown of sociodemographic characteristics, where possible and sample size permitting. Ideally, outcomes are also reported by sociodemographic groups of interest, or included as covariates in models to better understand their potential impact on or association with outcomes (see, eg, [94]). However, given the small sample sizes in many sociodemographic subgroups of interest, traditional hypothesis-confirming statistical methods of significance testing and modeling may not be possible. For such cases, we suggest focusing on exploratory, descriptive, and hypothesis-generating approaches such as within-group effect sizes (eg, the effect size of a symptom change score in non–Hispanic Black participants) and between-group effect sizes (eg, the effect size of the difference between symptom change scores in non–Hispanic Black and non–Hispanic White participants) where possible. Consolidating certain subgroups into another broader group may also be necessary in certain cases (eg, collapsing “genderqueer,” “nonbinary,” “agender,” and “different identity” into “other” for gender identity; see [95]), ideally to reach a sample size of at least 20-25 per subgroup [96], although greater care must be taken in the interpretation of these less precise groupings. For this exploratory approach, replication is key to identifying consistent and reliable patterns, and these analyses should be prespecified and conducted for every study even if only for internal purposes. Data from these exploratory analyses can be used to identify potential questions and hypotheses for future research, provide preliminary data for power analyses and study planning, and inform intervention improvements and possible precision targets. Researchers should also go beyond the assessment of differences in outcomes by sociodemographic characteristics, and consider how they can identify, capture, and analyze the structural factors (eg, discrimination, exclusionary policies, digital redlining) that may be responsible for creating or exacerbating inequities [97,98].

The outcomes assessed should be comprehensive and include efficacy (eg, symptom change), engagement (eg, app use metrics, therapeutic alliance), satisfaction and feasibility (including trust and perception of bias), and safety. Moreover, the assessed outcomes must reflect not only the priorities and values of the researchers, but also those of the participants. Doing so allows for a more nuanced understanding of the impact of DMHIs on various groups of people, and is necessary for a precision-based research and development approach that can be leveraged to address health equity and create better products. Qualitative data (eg, free-text questions about app experience, participant interviews) should be solicited, as they illuminate the nuance of experience across different groups of people and can reveal previously unknown issues or concerns among users.

Safety, in particular, is an important outcome to examine in the context of health equity [99]. Despite this, not all DMHI trials provide safety data and there is little standardization in how adverse events are captured and reported [100]. This may be, to a certain extent, because different types of DMHIs require varying degrees of safety capturing and oversight. Trials also vary considerably in the level of clinical support to mitigate and manage potential adverse events. Safety measurement must align with each intervention’s risk and capabilities to address safety concerns, while being as consistent as possible across DMHIs. Lastly, when generating internal safety reports for each trial, safety events and any other related data should be provided alongside sociodemographic data to allow exploration of prevalence rates within different groups of people.

All of these equity-centering research practices, from recruitment and data collection to analysis and safety considerations, should also be integrated into iterative internal product development processes and user experience (UX) research as well as external evidence-generation efforts.

## Advocacy, Policy, Regulation, and Funding

Shifting our view “up” to another level, we turn to considerations of advocacy, policy, regulation, and funding, which may unlock paths to address inequities unavailable at the individual or even company level [27]. For example, the translation of mental health evidence–generation findings into new policies intended to improve health equity in mental health care has been bolstered by the implementation of advocacy efforts and new policy, regulation, and funding efforts at both the federal and local levels [52,101,102]. Advocacy efforts such as lobbying and grassroots campaigns focused on improving the equitable distribution of mental health services and awareness of mental health disparities have been critical in advancing policy change and allocation of resources as well [103,104].

In part due to these advocacy efforts, the past several decades have brought about various legislative initiatives focused on parity, community mental health initiatives, workforce diversity, and trauma-informed care that have served to improve mental health equity. Perhaps the most groundbreaking example of federal parity legislation in the past 25 years was the implementation of the Mental Health Parity and Addiction Equity Act of 2008 [105] which required insurance companies to offer mental health and substance use disorder care coverage similar to that of medical care, paving the way for equitable access to the support and services for those in need. Broader efforts to expand internet access have also evolved in recent years. These initiatives include broadband expansion that uses public or private funding to build the infrastructure needed to deliver internet to areas without current capabilities [106], net neutrality regulations established in 2003 [107], public Wi-Fi funded by governments or municipalities, and digital literacy programs that teach the fundamentals of using the internet [108]. The ability of users to connect online to DMHIs has been greatly enhanced by these collective efforts.

The regulatory oversight of medical devices by the Food and Drug Administration (FDA) can also impact digital mental health equity. Clinical trial diversity initiatives, regulatory science research and policies, public health education initiatives, postmarket surveillance activities, and drug/device presubmission interactions and approval processes have the ability to support or leave vulnerable populations in need of additional digital mental health care [109-111]. For example, following the onset of the COVID-19 pandemic and the nationwide public health emergency, the FDA announced in April 2020 that it would temporarily waive certain requirements for digital therapeutics targeting psychiatric disorders. This measure aimed to provide urgently needed mental health support tools to a struggling population, although it expired in November 2023 [112].

In addition, various organizations such as the American Psychological Association (APA) [101], the National Institute of Mental Health (NIMH) [52], the US Preventive Services Taskforce (USPSTF) [37], the Substance Abuse and Mental Health Services Administration (SAMHSA) [102], and others have provided support for recommendations and funding that have advanced mental health equity. For example, the USPSTF recently developed a framework to incorporate health equity into each step of their recommendation-making process. The NIMH’s ongoing Laboratories to Optimize Digital Health program seeks to fund innovative research projects that study ways to increase access, efficacy and effectiveness, and quality of DMHIs, particularly among those who experience health disparities [113]. Beyond the critical research focused on interventions to help more vulnerable populations, researchers should also focus on understanding individual-level differences in the prevalence, root causes, and mechanisms of expression of different mental health challenges [114-116]. Additional funding opportunities should be made available to train underrepresented minorities, develop culturally competent interventions, and determine the impact of the implementation of interventions or policies within underserved communities and health care settings. Trends in research that require the involvement of patients with lived experiences, interventions integrating peer support, and community-based participatory research [63] highlight the importance of including multiple stakeholders to identify priorities, develop and test interventions, and inform policy. These efforts reflect a commitment to advocating for and enacting policies that guide, regulate, and fund work that ensures a wide variety of users can access and benefit from DMHIs. Progress in these areas will be crucial in advancing equity across the field of DMHI in the future.

## Commercial and Market Engagement Strategy

For-profit companies and commercial endeavors represent an opportunity to accelerate the traction of equity-related efforts in mental health. Company success in terms of market penetration and financial growth are well aligned and in many cases dependent on the factors described earlier (DEIB maturity in the workforce, more inclusive practices in product design and evidence generation, more representative and true to real-world evidence gathering, and reinforcement of equity-related policy). Today’s market rewards and reinforces equity-centered practices as customers are seeking products that meet sophisticated requirements in this regard. Deloitte estimated that their life sciences clients could garner a 10× return on their investment by bolstering equitable care access in underserved markets [38] and entrepreneurs focused on mental health equity are attracting early support and funding [117]. That said, challenges exist for for-profit ventures as well. For example, the cost of providing DMHI access to “hard to reach” populations (eg, those without a smartphone or internet access, or who are members of groups too small to amass commercial attention) may be greater, impacting profitability and sustainability of outreach efforts (see Galea [118] for an analogous case related to the greater cost of vaccinating certain vulnerable populations). Moreover, in cases where end users, and not an intermediary organization (eg, an employer, health plan, or health care provider), directly bear some or all of the cost for access to a DMHI, existing financial inequities may perpetuate or exacerbate inequities in mental health access and outcomes [119]. In both these cases, commercial stakeholders may consider steps such as advocating for ring-fenced funding, or allocation of protected funds that are specifically and exclusively intended for the purpose of reaching historically minoritized and underserved populations. Importantly, the purpose of these protected funds would not be to optimize customer acquisition costs, but rather to optimize representation.

Commercial and medical affairs functions in health care organizations such as DMHI and pharmaceutical manufacturers play a vital role in translating and reinforcing progress from science/policy out to the market and market demands back to internal groups [120]. Customer listening allows firms to collect very clear requirements on how products need to function and be deployed to address real-world equity-related challenges, helping product and evidence teams prioritize their road maps [39]. In the other direction, commercial and medical affairs teams showcase emerging work by science and product, building confidence and momentum in the market to go after equity-related unmet needs with more urgency.

Strong and creative commercial and medical affairs functions can also help build differentiated paths to market by developing tailored marketing, enrollment, and payment models to meet the specific needs of underserved populations. Customers who struggle to meet quality standards or equity requirements may be more inclined to pursue digital solutions aggressively. Manufacturers can foster more effective deployments by offering configurable solutions and flexibility in implementation and marketing. Additionally, not all DMHI manufacturers operate as for-profit ventures; exploring other models for addressing health inequities, such as the US State Department’s “Public Health 3.0” initiative [121] and nonprofit business models [122], is crucial for scaling new products.

## Equity-Informed Implementation Science in Real-World Deployments

Although it appears as the final section of this paper, the importance of thoughtful consideration of health equity within and across DMHI ecosystem deployments cannot be underscored enough. This view is critical because it spans the individual, familial, and community levels, as well as the larger encompassing “ecosystem” organizations, such as health care systems or provider networks, which may be in unique positions to operate across multiple levels of SDOH to address inequities [27]. Deployment is also where “the rubber meets the road,” and the actual impact of DMHIs is realized.

Implementation science frameworks offer considerations into how evidence-supported interventions are “implemented” in routine practice in naturalistic, real-world contexts; and may, for example, recommend evaluation of factors such as immediate and sustained adoption of the intervention, cost, and barriers therein [123]. Such real-world data are of paramount importance in determining not only the real-world effectiveness of DMHIs, but also integral for informing and refining approaches to deployment processes (ie, provider training and patient-facing information). Moreover, such data create an opportunity for monitoring if the DHMI or its deployment characteristics are closing or exacerbating equity gaps, paving the way for deployment adjustments accordingly.

Ecosystems purchasing or deploying DMHIs (eg, payer, provider, value-based care organizations) are encouraged to transparently and reliably emphasize the need for health equity to be included in a manufacturer’s DMHI product and evidence-generation portfolio before adoption or deployment within their system. To begin, existing implementation science frameworks have been adapted to focus on health equity and can provide thoughtful questions for ecosystems and deployment partners to consider. These may include an early focus on “reach” (ie, who is included and excluded in different deployment strategies), proactive design and selection of interventions for vulnerable populations, and measuring and monitoring outcomes through the lens of addressing inequities [40]. Ecosystems may also consider adopting a health equity–informed implementation science framework [33], such as the Evidence- and Consensus-Based Digital Healthcare Equity Framework [41], or may choose to create and implement their own, as demonstrated by health care organizations such as Elevance [124], Kaiser Permanente [125], Reliant Medical Group [126], and the Centers for Medicare and Medicaid Services [127].

One example of how health equity might be considered in the deployment of a DMHI within a health care ecosystem is through the collection, monitoring, and evaluation of SDOH data. DMHIs (or their underlying platform infrastructure) could be designed to thoughtfully gather information about specific SDOH factors from patients. Such data could inform further risk assessment or clinical follow-ups, and facilitate the offering of available resources provided by the system to the patient. Processes like this may enable earlier intervention, more precise tailoring of interventions, and greater utilization of available support and resources for those in greatest need. This approach has the potential to benefit underserved individuals, DMHI manufacturers, and health care systems as the shift toward whole-person care evolves.

New models of care that support DMHI implementation are also emerging in postpandemic health care delivery: one such role is the digital navigator. This role facilitated the rapid transition to telehealth in the early days of the pandemic as a means to support patients accessing platforms to access care. The digital navigator can access which phone and data plan a patient has to optimally match them with a DMHI that takes the whole-person’s life context into account. This role is one method for addressing the friction from referral to activation that is often experienced in real-world deployments [128]. Other promising examples of alternative care models are stepped care [129,130] and collaborative care [131,132], both of which have accrued growing bodies of support in the literature, and which offer opportunities for DMHI integration and implementation. As these new models of care mature, data exchange to support the optimal levels of human and digital touchpoints can support the broader use of DMHIs in populations that may not traditionally seek care for mental health.

## Conclusions

The potential to advance health equity through innovation in DMHIs is apparent, but by no means guaranteed. Here, utilizing decades of learning across multiple domains of expertise, we explored the progress and opportunities within the DMHI field to address mental health care inequities and articulated several steps essential to mobilize and sustain meaningful forward progression in this endeavor. While notable progress has been made over the past 25 years, we advocate for the field’s thoughtful and evergreen advancement in inclusivity and equity, thereby continuing to redefine the mental health care experience for this generation and those to come.

## Abbreviations

AI: artificial intelligence
APA: American Psychological Association
DEIB: diversity, equity, inclusion, and belonging
DMHI: digital mental health intervention
FDA: Food and Drug Administration
GDPR: General Data Protection Regulation
LGBTQIA+: Lesbian, Gay, Bisexual, Transgender, Queer, Intersex, Asexual+
LLM: large language model
ML: machine learning
NIH: National Institutes of Health
NIMH: National Institute of Mental Health
PIDAR: Partner, Identify, Demonstrate, Access, Report
REACH: Rush Education and Career Center Hub
RE-AIM: Reach, Effectiveness, Adoption, Implementation, Maintenance
SAMHSA: Substance Abuse and Mental Health Services Administration
SDOH: social determinants of health
STEM: Science, Technology, Engineering, Mathematics
USPSTF: US Preventive Services Taskforce
